# Supplementary material for: Hybrid Models and Biological Model Reduction with PyDSTool
Source: PLoS Comput Biol. 2012 Aug 9;8(8):e1002628. doi: 10.1371/journal.pcbi.1002628 (PMC3415397; doi:10.1371/journal.pcbi.1002628)
Supplement: Text S4 — Complete source code for the PyDSTool package (version 0.88.120504). Includes API documentation and help files linking to web pages. This file is identical to the current public release on Sourceforge.net. (ZIP) [file pcbi.1002628.s004.zip › PyDSTool/html/toc-matplotlib.pylab-module.html]

xml version="1.0" encoding="ascii"?


pylab


# Module pylab

---

## Functions

beta  
 binomial  
 bytes  
 chisquare  
 exponential  
 f  
 gamma  
 geometric  
 get\_state  
 gumbel  
 hypergeometric  
 laplace  
 load  
 logistic  
 lognormal  
 logseries  
 multinomial  
 multivariate\_normal  
 negative\_binomial  
 noncentral\_chisquare  
 noncentral\_f  
 normal  
 pareto  
 permutation  
 poisson  
 power  
 rand  
 randint  
 randn  
 random  
 random\_integers  
 random\_sample  
 ranf  
 rayleigh  
 sample  
 save  
 seed  
 set\_state  
 shuffle  
 standard\_cauchy  
 standard\_exponential  
 standard\_gamma  
 standard\_normal  
 standard\_t  
 triangular  
 uniform  
 vonmises  
 wald  
 weibull  
 zipf  

## Variables

ALLOW\_THREADS  
 BUFSIZE  
 CLIP  
 DAILY  
 ERR\_CALL  
 ERR\_DEFAULT  
 ERR\_DEFAULT2  
 ERR\_IGNORE  
 ERR\_LOG  
 ERR\_PRINT  
 ERR\_RAISE  
 ERR\_WARN  
 FLOATING\_POINT\_SUPPORT  
 FPE\_DIVIDEBYZERO  
 FPE\_INVALID  
 FPE\_OVERFLOW  
 FPE\_UNDERFLOW  
 FR  
 False\_  
 HOURLY  
 Inf  
 Infinity  
 MAXDIMS  
 MINUTELY  
 MO  
 MONTHLY  
 NAN  
 NINF  
 NZERO  
 NaN  
 PINF  
 PZERO  
 RAISE  
 SA  
 SECONDLY  
 SHIFT\_DIVIDEBYZERO  
 SHIFT\_INVALID  
 SHIFT\_OVERFLOW  
 SHIFT\_UNDERFLOW  
 SU  
 ScalarType  
 TH  
 TU  
 True\_  
 UFUNC\_BUFSIZE\_DEFAULT  
 UFUNC\_PYVALS\_NAME  
 WE  
 WEEKLY  
 WRAP  
 YEARLY  
 absolute  
 add  
 arccos  
 arccosh  
 arcsin  
 arcsinh  
 arctan  
 arctan2  
 arctanh  
 bitwise\_and  
 bitwise\_not  
 bitwise\_or  
 bitwise\_xor  
 c\_  
 cast  
 ceil  
 colorbar\_doc  
 conj  
 conjugate  
 copysign  
 cos  
 cosh  
 deg2rad  
 degrees  
 divide  
 e  
 equal  
 exp  
 exp2  
 expm1  
 fabs  
 floor  
 floor\_divide  
 fmax  
 fmin  
 fmod  
 frexp  
 greater  
 greater\_equal  
 hypot  
 index\_exp  
 inf  
 infty  
 invert  
 isfinite  
 isinf  
 isnan  
 ldexp  
 left\_shift  
 less  
 less\_equal  
 little\_endian  
 log  
 log10  
 log1p  
 logaddexp  
 logaddexp2  
 logical\_and  
 logical\_not  
 logical\_or  
 logical\_xor  
 maximum  
 mgrid  
 minimum  
 mod  
 modf  
 multiply  
 nan  
 nbytes  
 negative  
 newaxis  
 nextafter  
 not\_equal  
 ogrid  
 ones\_like  
 pi  
 r\_  
 rad2deg  
 radians  
 rcParams  
 rcParamsDefault  
 reciprocal  
 remainder  
 right\_shift  
 rint  
 s\_  
 sctypeDict  
 sctypeNA  
 sctypes  
 sign  
 signbit  
 sin  
 sinh  
 spacing  
 sqrt  
 square  
 subtract  
 tan  
 tanh  
 true\_divide  
 trunc  
 typeDict  
 typeNA  
 typecodes  

---

[hide private]
